# World Trade Center Disaster: A Qualitative Study of Older Enrollees in the World Trade Center Health Registry

**DOI:** 10.1093/geroni/igaf122.2969

**Published:** 2025-12-31

**Authors:** Robert Brackbill, Alexis Merdjanoff, Lydia Leon, Janna Metzler, Felix Ortega, Ashley Rojas, Ndidi Umezinwa

**Affiliations:** New York City Department of Health and Mental Hygiene, Long Island City, New York, United States; New York University, New York, New York, United States; New York City Department of Health and Mental Hygiene, Long Island City, New York, United States; New York City Department of Health and Mental Hygiene, Long Island City, New York, United States; New York City Department of Health and Mental Hygiene, Long Island City, New York, United States; New York City Department of Health and Mental Hygiiene, Long Island City, New York, United States; New York City Department of Health and Mental Hygiene, Long Island City, New York, United States

## Abstract

The World Trade Center disaster on September 11, 2001 has had long-lasting effects on the health and quality of life of those exposed to the disaster, including lung problems, GERD, cardiovascular diseases, PTSD, depression and other ailments that are likely to worsen with age. The World Trade Center Health Registry, a longitudinal exposure cohort of 71,436 persons who were working or living in the impacted area, has embarked on a mixed model study of older persons in the Registry (55+) that includes a qualitative study followed by survey-based data collection, and in-person cognitive assessment. Recruitment for the qualitative study consisted of a random sample of enrollees 55 years and older stratified by age and sex with a cooperation rate of 67%. This presentation will describe the findings from 50 in-depth interviews with Registry enrollees that explored topics including activities and interpersonal relationships; current health and cognitive issues; stress and well-being; quality of life; the impact of 9/11 on aging; and the use of technology to support health. An overarching theme was that more than twenty years later these respondents continue to grapple with their 9/11 experience. For many, 9/11 was a key turning point in their lives that influenced how they approach growing older; their relationships with others; their own mortality; and, their concerns for their health in the future. This study further supports the need for long-term data collection and analysis of exposed cohorts following a collective trauma like 9/11, especially as the populations grow older.

